# *Echinococcus multilocularis* and *Echinococcus granulosus* in canines in North-Khorasan Province, northeastern Iran, identified using morphology and genetic characterization of mitochondrial DNA

**DOI:** 10.1186/s13071-019-3859-z

**Published:** 2019-12-27

**Authors:** Zahra Heidari, Mitra Sharbatkhori, Iraj Mobedi, Seyed Hossein Mirhendi, Bahram Nikmanesh, Meysam Sharifdini, Mehdi Mohebali, Zabihollah Zarei, Kourosh Arzamani, Eshrat Beigom Kia

**Affiliations:** 1 Department of Medical Parasitology and Mycology, School of Public Health, Tehran University of Medical Sciences, Tehran, Iran; 2 Center for Research of Endemic Parasites of Iran (CREPI), Tehran University of Medical Sciences, Tehran, Iran; 3 Infectious Diseases Research Center, Golestan University of Medical Sciences, Gorgan, Iran; 4 Department of Medical Parasitology and Mycology, School of Medicine, Isfahan University of Medical Sciences, Isfahan, Iran; 5 Department of Lab Medical Sciences, School of Allied Medical Sciences, Tehran University of Medical Sciences, Tehran, Iran; 6 Department of Medical Parasitology and Mycology, School of Medicine, Guilan University of Medical Sciences, Rasht, Iran; 7 Vector-borne Diseases Research Center, North Khorasan University of Medical Sciences, Bojnurd, Iran

**Keywords:** Canine, *Echinococcus granulosus*, *Echinococcus multilocularis*, Mitochondrial genes, Morphology, Iran

## Abstract

**Background:**

Canids are definitive hosts of *Echinococcus multilocularis* and *Echinococcus granulosus.* This study aimed to survey these two *Echinococcus* species in canids of North-Khorasan Province, northeastern Iran, using morphological criteria and genetic characterization of mitochondrial DNA.

**Methods:**

The carcasses of 106 canids, namely 61 jackals (*Canis aureus*), 23 foxes (*Vulpes vulpes*), 19 dogs (*Canis familiaris*) and three wolves (*Canis lupus*) were collected from the study area in 2013–2014 and examined for *Echinococcus* species. Morphological features were assessed by microscopy of adult worms. For molecular characterization, DNA was extracted, mostly from the adult worms but also from eggs. DNA fragments of the cytochrome *c* oxidase subunit 1 (*cox*1) and NADH dehydrogenase subunit 1 (*nad*1) mitochondrial genes were amplified and sequenced. Sequences were aligned and compared with reference sequences. Intraspecific and interspecific diversity were calculated and phylogenetic analysis was performed.

**Results:**

Overall, 9.4% of the canids (eight jackals and two foxes) were found infected with *E. multilocularis* by molecular methods, of which seven cases were also confirmed using morphological description of the adult worms. *Echinococcus granulosus* was found in 6.6% of the canines (four dogs, two jackals and one wolf) as determined by both molecular methods and adult cestode morphology. All *E. granulosus* isolates were identified as the G1 genotype. Comparative sequence analysis indicated 0–0.7% and 0% intraspecific divergence within *E. granulosus* isolates and 0% and 0–0.2% within *E. multilocularis* isolates for *cox*1 and *nad*1, respectively.

**Conclusions:**

This study revealed the presence of *E. multilocularis* and *E. granulosus* in canids of North-Khorasan Province of Iran. Jackals were found infected with both *E. multilocularis* and *E. granulosus*, but infection with the former species was higher.
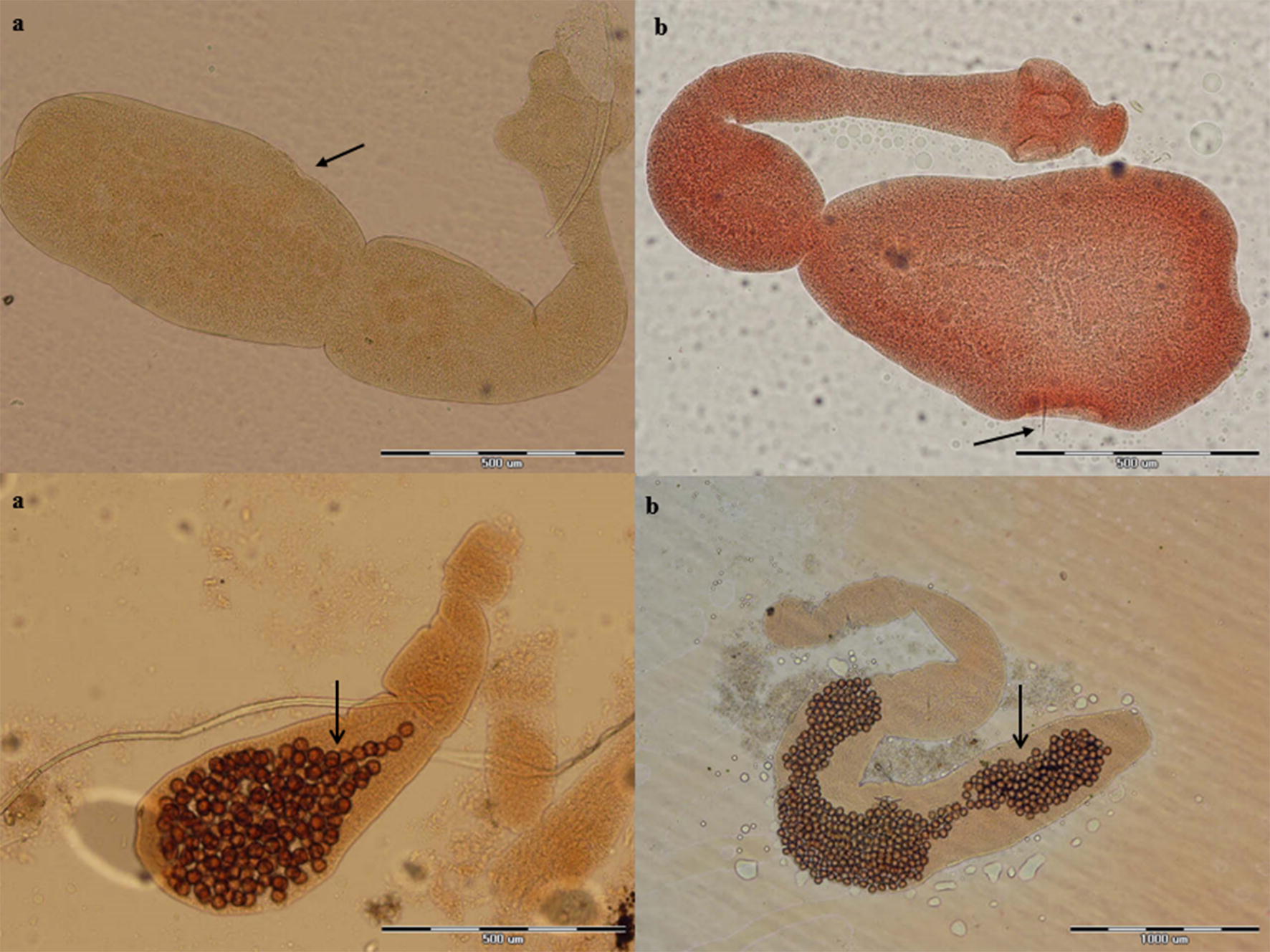

## Background

Echinococcosis, caused by the larval stage of tapeworms of the genus *Echinococcus*, is one of the most important zoonotic diseases worldwide [1]. *Echinococcus granulosus* (*sensu lato*) and *Echinococcus multilocularis* are the most prevalent species infecting humans, resulting in cystic echinococcosis (CE) and alveolar echinococcosis (AE), respectively. *Echinococcus granulosus* (*s.l.*) is known to be endemic in all continents, while *E. multilocularis* has a more restricted distribution, generally regarded a parasite limited to the northern hemisphere [2]. Both AE and CE are considered neglected zoonoses, with a global distribution and higher prevalence for CE, but a higher pathogenicity and mortality for AE, especially in Asia [3]. Herbivores are intermediate hosts for *E. granulosus* (*s.l.*), and canids, including dogs, wolves, foxes and jackals, act as definitive hosts, harboring the adult worms in the villi of the small intestine [4]. The life-cycle of *E. multilocularis* involves several carnivores such as foxes, coyotes, dogs and cats as definitive hosts, and rodents as intermediate hosts. Humans can be an accidental dead-end intermediate host for both species *via* close contact with the definitive host or by indirect ingestion of eggs through contaminated water and uncooked food [5].

Without data on genetic variation within and between populations of *Echinococcus*, no decisions can be made about breeding systems, extent of gene flow, species delineation or modes of speciation [6]. During the past decades, molecular studies, mainly based on mitochondrial genes, have described several genotypes or species within *E. granulosus* (*s.l.*), revealing a species complex as follows: *E. granulosus* (*sensu stricto*) (genotypes G1–G3), *E. equinus* (G4), *E. ortleppi* (G5), *E. canadensis* (G6–G10) and *E. felidis* (‘lion strain’); the existence of the human-specific genotype G9 is controversial [1, 7]. Recently, Kinkar et al. [8] showed that G1 and G3 are two distinct mitochondrial genotypes and can be considered as a single species of *E. granulosus* (*s.s.*), whereas G2 is not a separate genotype but belongs to G3. They suggested eliminating G2 from the list of *E. granulosus* genotypes. Laurimae et al. [9] confirmed that based on six nuclear loci, G6/G7 and G8/G10 genotypes can be considered as two distinct species. Additionally, Thompson [10] proposed to consider camel and pig strains of *E. granulosus* as a single species (*E. intermedius*) as originally suggested by Lopez-Neyra & Soler Planas in 1943 [11]. Unlike *E. granulosus* (*s.l.*), only minor variations have been detected in the cytochrome *c* oxidase subunit 1 (*cox*1) and NADH dehydrogenase subunit 1 (*nad*1) mitochondrial DNA sequences of *E. multilocularis* isolates, resulting in the recognition of two groups, namely M1 and M2. M1 originates in China and North America and M2 in Europe [12, 13].

To date, molecular studies on *E. granulosus* carried out in Iran have reported several genotypes in domestic livestock (genotypes G1–G7) [14–18] as well as in humans (G1–G3 and G6) from different endemic foci of Iran [19–23]. Additionally, the genotypes G1 [24–27], G2 [24], G3 [24–26] and G6 [25] have been identified in dogs in Iran.

So far, several documented human cases of alveolar echinococcosis have been reported in different parts of Iran [28–30]. Canine infection with adult worms of *E. multilocularis* has been previously reported in red foxes [31, 32] and jackals [32] from northwestern Iran based on morphological criteria. Furthermore, *E. multilocularis* has been reported in carnivores from the Razavi Khorasan Province, northeastern Iran, using analysis of *nad*1 mitochondrial DNA [33].

In Iran, domestic dogs, but also wild canids including foxes, jackals and wolves, are known as important reservoirs of echinococcosis [34]. However, information on the role of such animals in the spread of this disease is available for only some provinces. The North-Khorasan Province, located in northeastern Iran, is bordering with Turkmenistan where *E. multilocularis* is believed to be endemic [35]. There are reports of human CE in North-Khorasan Province [36–38]. There are also reports of *E. multilocularis* in carnivores in Chenaran City in the adjacent province, Razavi Khorasan [33]. Additionally, a variety of rodent species, including the family Cricetidae, members of which act as the main natural intermediate hosts of AE in other endemic parts of the world [39], have also been observed in northeastern Iran [40]. Nevertheless, at present there are no data available on *Echinococcus* spp. in the canids of this province. Therefore, the purpose of this study was to identify species and genotypes of *Echinococcus* spp. in canids of North-Khorasan Province using morphological criteria and sequencing partial *cox*1 and *nad*1 genes.

## Methods

### Study area

**Fig. 1:**
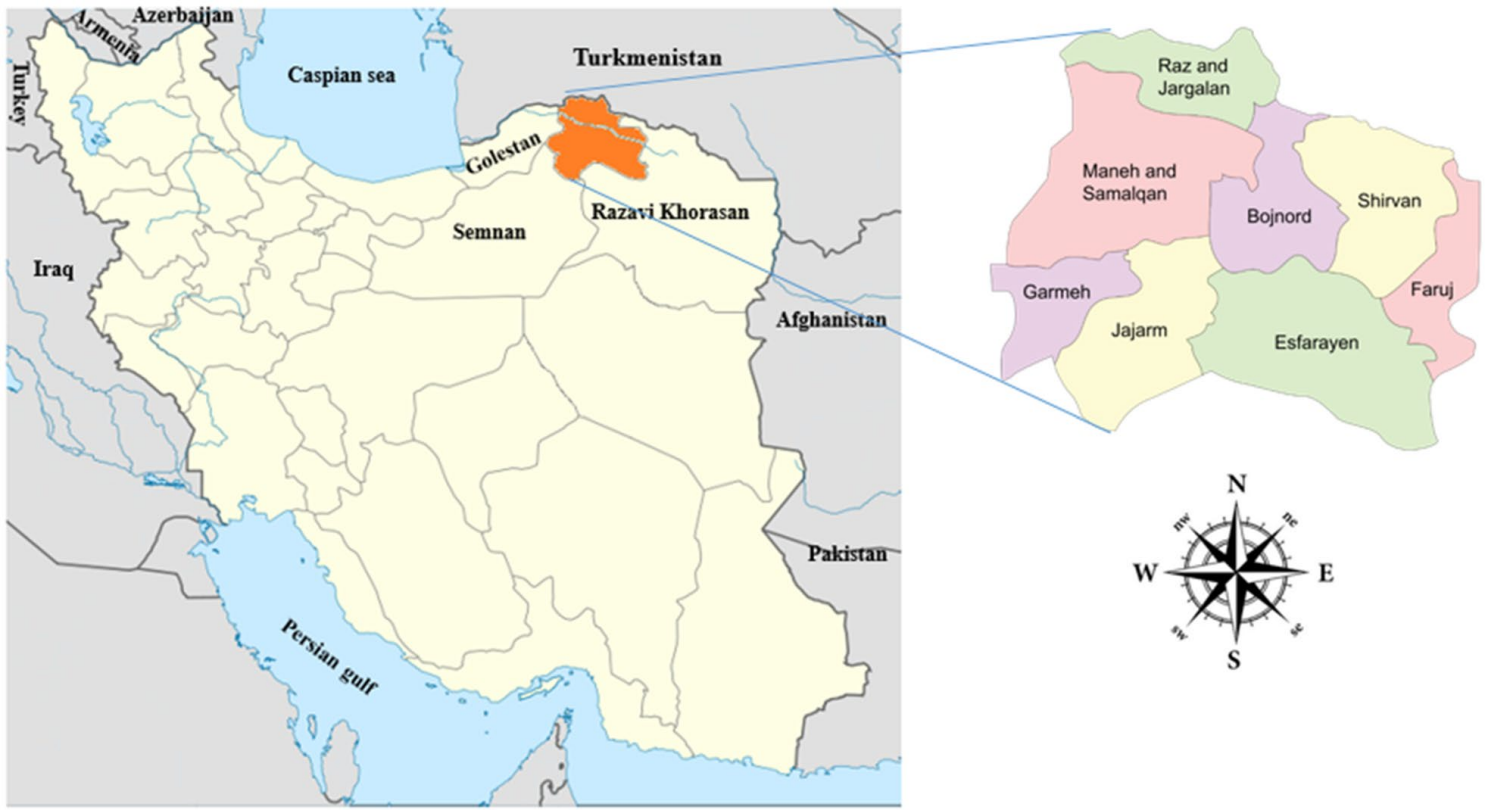
Left: map of Iran highlighting the study area, Northern Khorasan Province, and location of neighboring countries and provinces. Right: map of Northern Khorasan Province showing the capital, Bojnord, and different counties of the province

North-Khorasan Province is located between 36°37′–38°17′ N and 55°53′–58°20′ E, comprising an area of 28,434 km^2^ in northeastern Iran and sharing a border with Turkmenistan (Fig. 1). The capital is Bojnord. The province has a temperate climate with cold winters. The variety of different climates in this province is because of the vast mountains and forests. This region is geographically divided into two parts: mountain areas and lowland areas.

### Sampling

Study samples comprised carcasses of 106 canids [61 golden jackals (*Canis aureus*); 23 red foxes (*Vulpes vulpes*); 19 dogs (*Canis familiaris*); and three gray wolves (*Canis lupus*)] provided by the Vector-Borne Diseases Research Center (VDRC) in Bojnord. The carcasses were collected from 2013 to 2014 and reflected mostly road-kill accidents in different locations of the province. Other carcasses were part of the collection from other research projects in the same area on visceral leishmaniasis in wild canines, in collaboration with the VDRC [41].

In the VDRC, a veterinary practitioner recorded the characteristics of each animal including age group and sex in the relevant registration form with a specific identification code. The approximate age of the animals was determined according to the shape and color of teeth and dental formula to three groups (cub, under 2 years; young, 2–5 years; adult, > 5 years).

Under safety precautions, every carcass was dissected, and the entire length of the small intestine tied off, removed and stored in plastic container in a mixture of 70% ethanol (ethanol and water, 70:30, v/v). Fecal samples were also prepared from the rectum of the canids and kept in separate plastic containers with 70% ethanol. All samples were transported to the School of Public Health, Tehran University of Medical Sciences, Iran, and stored at − 20 °C for at least one month prior to examination.

### Parasitological examinations

For examination of the small intestines, the intestinal scraping technique (IST) was applied under safety precautions as described by Deplazes & Eckert [42]. In brief, each small intestine was opened along its full length in a metal tray, and after removal of coarse materials and large helminths, deep mucosal scrapings were made from the proximal, middle and posterior third of the small intestine using microscopic slides. The adhering materials were transferred to plastic Petri dishes and examined for adult *Echinococcus* tapeworms under a stereomicroscope. Additionally, small intestinal content and the remaining mucosa were washed with water through a sieve and the collected precipitations were examined in the same way for detection of *Echinococcus*. After detection, adult worms were removed, and for each intestine, ten worms were prepared for morphological diagnosis, after clearing in lactophenol and temporary staining using FAAL stain (formalin, alcohol, azocarmine and lactophenol) [43].

To detect taeniid eggs in feces, all fecal samples were examined by the formalin-ether sedimentation technique [44]. If an animal was negative for adult Taeniidae (*Echinococcus* spp. or *Taenia* spp.) by IST, but stool-positive for taeniid eggs, the correspondent fecal sample was later processed for molecular analysis.

The morphological characteristics of *Echinococcus* adult worms were studied using a calibrated microscope with consideration of specific criteria including body length, appearance of rostellar hooks (shape, total length and blade length of large and small hooks), and features of the strobila such as the position of genital pore in proglottids and the shape of the uterus. Species identification was performed according to published guidelines [45, 46]. One adult worm per intestine was kept in 70% ethanol at − 20 °C for further molecular analysis.

### Molecular analysis

#### DNA extraction

For each canid from which adult *Echinococcus* spp. were recovered by intestinal examination, genomic DNA was extracted from a single morphologically identified worm. Additionally, a DNA extraction protocol was performed on those fecal samples in which taeniid eggs were detected but where no adult taeniid worms could be found in the intestine.

After removal of the ethanol from adult *Echinococcus* worms and from fecal samples, these were washed twice with sterile distilled water, and total genomic DNA was extracted using a Bioneer tissue DNA extraction kit and stool DNA extraction kit (Bioneer, Daejeon, South Korea), respectively, according to manufacturer’s instructions. All extracted DNA was stored at − 20 °C until PCR amplification.

#### Polymerase chain reaction (PCR)

Genomic DNA samples were analyzed using amplification of mitochondrial DNA within the cytochrome *c* oxidase subunit 1 (*cox*1) and NADH dehydrogenase subunit 1 (*nad*1) genes, separately. All PCR reactions were carried out in a final volume of 25 μl, consisting of 12.5 μl of master mix (2× Master Mix RED; Ampliqon, Odense, Denmark; 1.25 U Taq DNA polymerase, 0.5 μM of dNTPs and 2 mM MgCl_2_), 20 pmol of each primer and 2 μl of DNA template. The forward and reverse primers used in the PCRs were as follows: JB3 (5′-TTT TTT GGG CAT CCT GAG GTT TAT-3′) and JB4.5 (5′-TAA AGA AAG AAC ATA ATG AAA ATG-3′) for the *cox*1 gene [12] and JB11 (5′-AGA TTC GTA AGG GGC CTA ATA-3′) and JB12 (5′-ACC ACT AAC TAA TTC ACT TTC-3′) for the *nad*1 gene [13]. The PCR program began with one cycle at 94 °C for 5 min (primary denaturation), followed by 35 cycles at 94 °C for 30 s (denaturation), 50 °C (*nad*1) or 55 °C (*cox*1) for 45 s (annealing) and 72 °C for 35 s (extension), followed by a final extension step at 72 °C for 10 min.

PCR products were electrophoresed on a 1.5% TBE (Tris 0.09 M, Borate 0.09 M, EDTA 0.02 M) agarose gel stained with Fluoro Dye Fluorescent DNA Loading Dye for loading and detecting DNA markers (SMOBiO DM3100; Bioshimigene, Tehran, Iran). Electrophoresis was carried out at 90 V for 45 min. PCR products were visualized using a UV transilluminator (Uvitec, Cambridge, UK) and digitally photographed. All PCR products of both *cox*1 and *nad*1 genes were purified with an AccuPrep Gel purification kit (Bioneer, Daejeon, South Korea) according to the manufacturer’s instructions.

#### Sequencing and phylogenetic analysis

Purified PCR products were first sequenced unidirectionally using the forward primers indicated above. After analysis of the results, if necessary, the process would be repeated to obtain desirable sequences. Sequences were edited and analyzed by Chromas software v.2.01 (Technelysium Pty Ltd., Brisbane, Queensland, Australia). Nucleotide sequences were compared with reference sequences in GenBank using the BLAST algorithm (http://www.ncbi.nlm.nih.gov/). In addition, sequences were trimmed, aligned using the software BioEdit v.7.0.9 [47], and compared with reference genotypes (G1–G10) of *E. granulosus* (*s.l.*), and species of *Echinococcus* from previous publications. Different nucleotide sequences of both *cox*1 and *nad*1 genes from this study were submitted to the GenBank database.

Phylogenetic analysis was performed of representative concatenated *cox*1 and *nad*1 DNA sequence data from the present study along with reference sequences of all known *E. granulosus* genotypes (G1–G10) and *Echinococcus* species, using *Taenia saginata* as the outgroup. The character-based Bayesian inference (BI) method was employed for the phylogenetic analyses using the software MrBayes v.3.1.2 [48]. Posterior probabilities (pp) were obtained for 1,000,000 generations (ngen: 1,000,000; ‛burn inʼ = 10,000). The program TreeviewX v.0.5.0 [49] was used to show the consensus tree.

## Results

### Parasitological and molecular findings

A total of 106 canid intestines were examined for infection with *Echinococcus* species by morphological and molecular methods. In Table 1, study animals have been listed according to sex and age group.

**Table 1:** Distribution of canines examined for infections by *Echinococcus* species in North-Khorasan Province, northeastern Iran, from 2013 to 2014 according to animal age and sex

Age group^a^	Male			Female			Total
	Cub	Young	Adult	Cub	Young	Adult	
Animal							
Jackal	5	35	7	2	9	3	61
Fox	3	12	–	–	7	1	23
Dog	3	13	2	1	–	–	19
Wolf	–	1	2	–	–	–	3
Total	11	61	11	3	16	4	106

^a^Cub, under 2 years; young, 2–5 years; adult, > 5 years

**Fig. 2:**
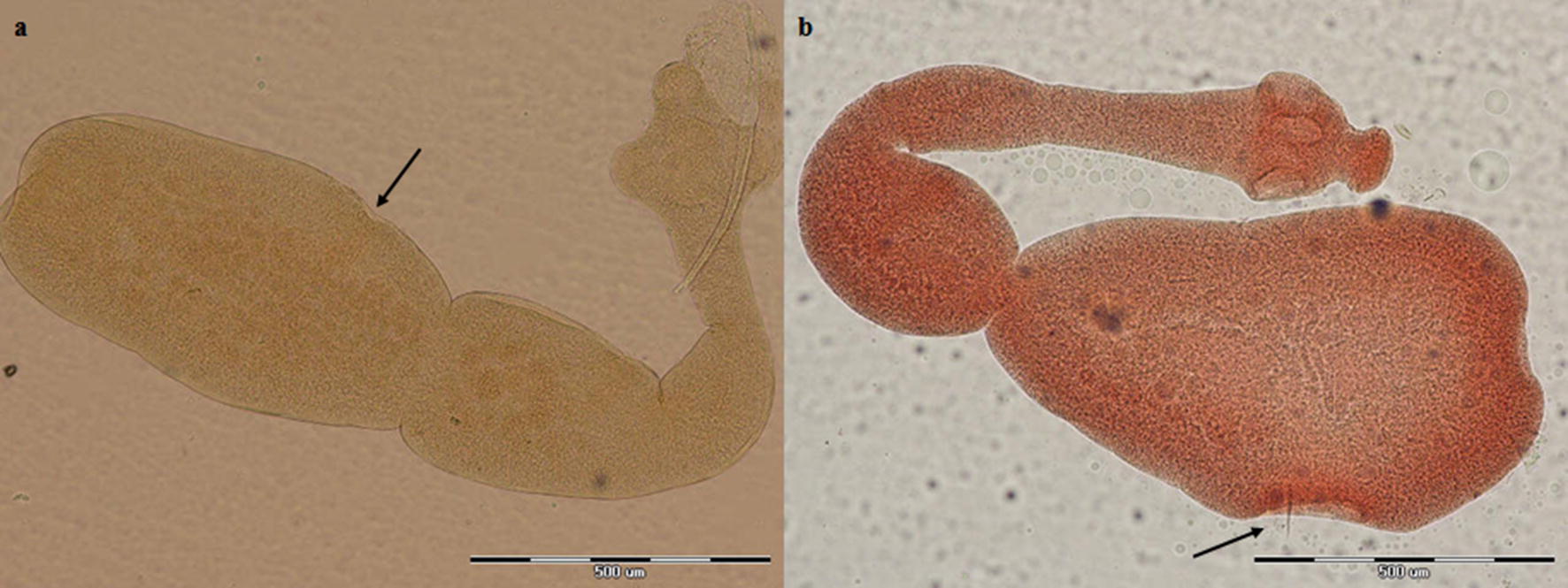
Photomicrographs of adult *Echinococcus multilocularis* (**a**) and *Echinococcus granulosus* (**b**) showing the position of the genital pore (arrow) in the gravid proglottid (anterior to mid-length in **a** and posterior to mid-length in **b**. *Scale-bars*: 500 μm

**Fig. 3:**
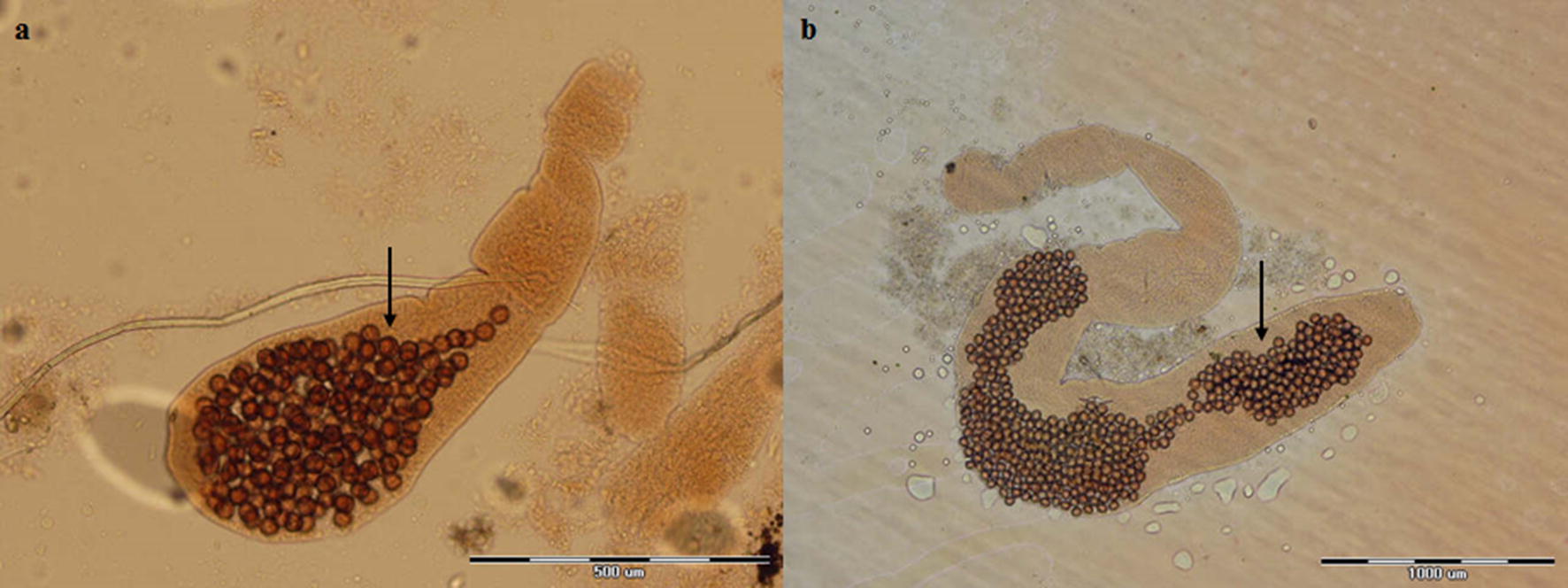
Photomicrographs of gravid proglottids of *Echinococcus multilocularis* (**a**) and *Echinococcus granulosus* (**b**) showing uterus (arrow) with eggs: a sac-like uterus (**a**) and laterally branching uterus (**b**). *Scale-bars*: **a**, 500 µm; **b**, 1000 μm

**Fig. 4:**
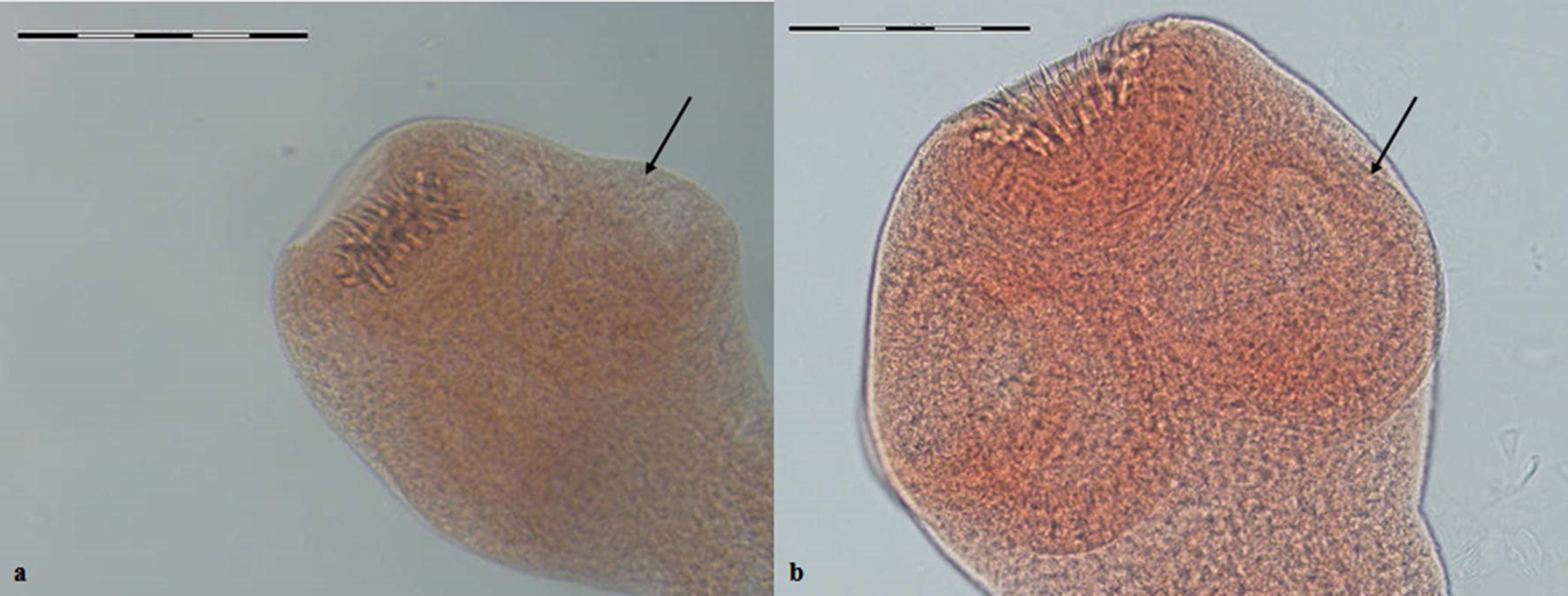
Photomicrographs of the scolex of *Echinococcus multilocularis* (**a**) and *Echinococcus granulosus* (**b**). Arrow indicates one sucker. *Scale-bars*: 100 μm

**Fig. 5:**
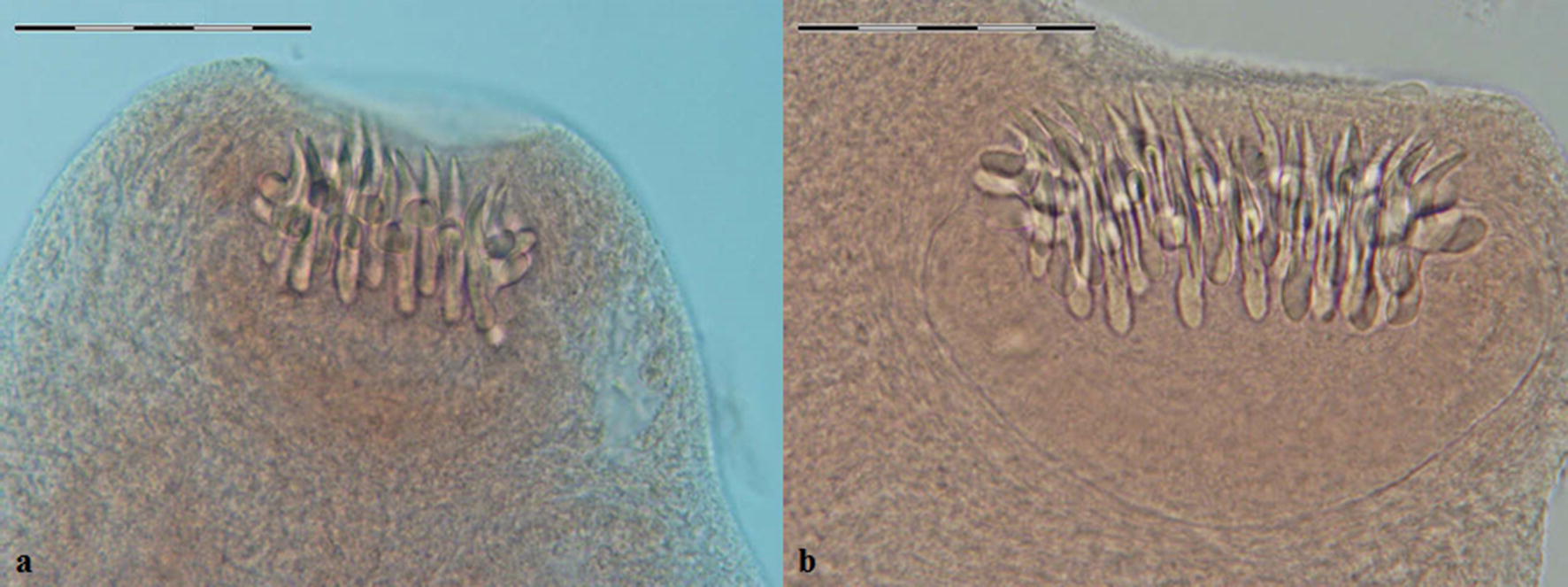
Photomicrographs of rostellar hooks of *Echinococcus multilocularis* (**a**) and *Echinococcus granulosus* (**b**). *Scale-bars*: 50 μm

**Fig. 6:**
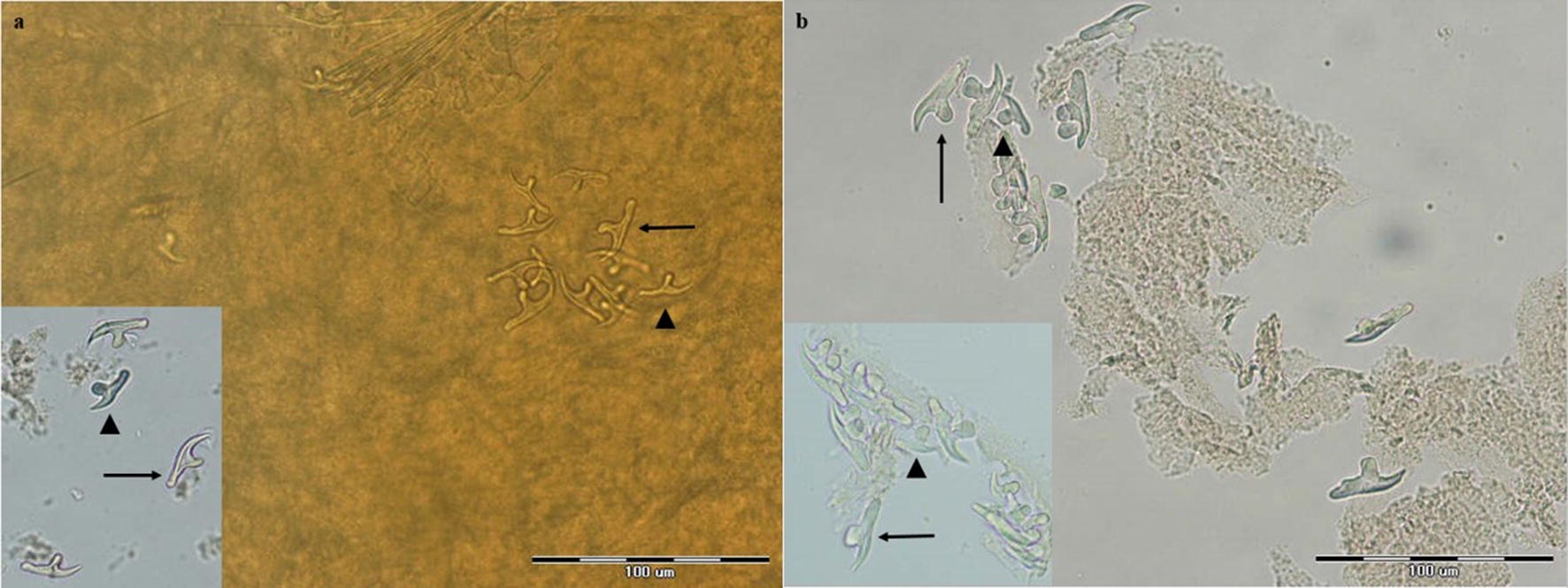
Photomicrographs of rostellar hooks of *Echinococcus multilocularis* (**a**) and *Echinococcus granulosus* (**b**): Arrow indicates a large hook and arrowhead indicates a small hook. *Scale-bars*: 100 μm

Overall, 17 of 106 canids (16%) were infected with *Echinococcus* species. Morphological species identification was carried out based on the characteristics of scolex and strobila. Figures 2, 3, 4, 5, 6 represent comparative morphological characteristics of *E. multilocularis* and *E. granulosus* as demonstrated by light microscopy. In the strobila, the total length and proglottid length of mature worms and position of the genital pore (Fig. 2), as well as the shape of the uterus in gravid proglottids (Fig. 3), were discriminative. However, in immature worms, there was an overlap in the size of strobila and proglottids. In the scolex, the dimensions of the suckers (Fig. 4) and of the large and small hooks as well as their shape (Figs. 5, 6) allowed the differentiation between *E. multilocularis* and *E. granulosus*. However, the number of hooks could not be counted accurately in all samples due to partial hook detachment during technical processing. Based on the morphology of adult worms, 14 animals were found infected with *Echinococcus* species: *E. multilocularis* (*n* = 7); and *E. granulosus* (*n* = 7) (Table 2). No co-infection with these species was found by morphological analysis of ten adult samples of *Echinococcus* from each infected animal.

For each canid from which adult *Echinococcus* was retrieved (*n* = 14), one morphologically described worm was processed for molecular analysis. Moreover, four fecal samples containing taeniid eggs for which corresponding intestinal examination failed to reveal any adult taeniids were also subjected to molecular investigation. Among these four samples, three cases were identified as *E. multilocularis* (Table 2) and the remaining one as *Taenia hydatigena*. Analysis of *cox*1 and *nad*1 genes of the adult *Echinococcus* spp. revealed the presence of both *E. granulosus* and *E. multilocularis* in the canids from the study area. In all mentioned *Echinococcus* isolates, fragments of approximately 450 and 500 bp were successfully amplified for *cox*1 and *nad*1 genes, respectively. Molecular identification based on both genes was in accordance with morphological identification of the adult worms.

**Table 2:** Morphological and molecular identification of *Echinococcus* isolates detected among different canines examined in North-Khorasan Province, northeastern Iran, according to animal age and sex

No.	Canid host			Echinococcus isolate		
	Species	Sex	Age group^a^	Morphology	Mitochondrial gene	
					cox1	nad1
1	Fox	Male	Young	E.m.	E.m.	E.m.
2	Fox	Male	Young	–^b^	E.m.	E.m.
3	Jackal	Male	Young	–^b^	E.m.	E.m.
4	Jackal	Male	Young	–^b^	E.m.	E.m.
5	Jackal	Male	Young	E.m.	E.m.	E.m.
6	Jackal	Male	Young	E.m.	E.m.	E.m.
7	Jackal	Male	Young	E.m.	E.m.	E.m.
8	Jackal	Male	Adult	E.m.	E.m.	E.m.
9	Jackal	Male	Adult	E.m.	E.m.	E.m.
10	Jackal	Male	Adult	E.m.	E.m.	E.m.
11	Jackal	Male	Young	E.g.	E.g. (G1)	E.g. (G1)
12	Jackal	Male	Adult	E.g.	E.g. (G1)	E.g. (G1)
13	Dog	Male	Cub	E.g.	E.g. (G1)	E.g. (G1)
14	Dog	Male	Young	E.g.	E.g. (G1)	E.g. (G1)
15	Dog	Male	Young	E.g.	E.g. (G1)	E.g. (G1)
16	Dog	Male	Young	E.g.	E.g. (G1)	E.g. (G1)
17	Wolf	Male	Young	E.g.	E.g. (G1)	E.g. (G1)

*Note*: Number of animals examined: 61 golden jackals (*Canis aureus*), 23 red foxes (*Vulpes vulpes*), 19 dogs (*Canis familiaris*) and 3 gray wolves (*Canis lupus*)

^a^Age groups: cub, under 2 years; young, 2–5 years; adult, > 5 years

^b^Due to severe autolysis of adult worms, diagnosis was performed only by molecular analysis of recovered eggs

*Abbreviations*: *E.m*., *Echinococcus multilocularis*; *E.g*., *Echinococcus granulosus*; G1, genotype G1

Among the 106 animals examined, *E. multilocularis* and *E. granulosus* were identified in ten and seven canids, respectively. All isolates of *E. granulosus* were identified as the G1 genotype. Table 2 summarizes molecular and morphological results for all *Echinococcus* isolates detected from different canines, according to sex and age group of the animals. The only animal species hosting both *E. multilocularis* and *E. granulosus* was the jackal; however, infectivity with *E. multilocularis* was higher (13.1 *vs* 3.3%). Among other canines, 8.7% of the foxes were found infected with *E. multilocularis*, while 21% of the dogs had *E. granulosus*. Finally, one out of three wolves was infected with *E. granulosus*. All infected animals were male animals (Table 2). However, it should be noticed that the sex balance of the study sample was skewed (83 males *vs* 23 females).

Among the *E. granulosus* isolates, two different *cox*1 sequences (scox1-1 and scox1-2) were found, while all *nad*1 sequences were identical (snad1-1). Among the *E. multilocularis* isolates, two different *nad*1 sequences (snad1-2 and snad1-3) were detected (intraspecific diversity of 0–0.2%), while all *cox*1 sequences were identical (scox1-3). Comparative sequence analysis showed 0–0.7% and 0% intraspecific genetic divergence within *E. granulosus* isolates and 0% and 0–0.2% within *E. multilocularis* isolates for *cox*1 and *nad*1, respectively. All 17 partial DNA sequences of *cox*1 and *nad*1 genes obtained in this study were deposited in the GenBank database under the accession numbers shown in Table 3.

**Table 3:** *Echinococcus multilocularis* and *E. granulosus* haplotypes from North-Khorasan Province, northeastern Iran, and origins of sequences used for concatenation (*cox*1 + *nad*1) in phylogenetic analyses (Fig. 2)

Representative haplotypes, genotypes and species	Host	*cox*1 (GenBank ID)	*nad*1 (GenBank ID)	Reference
**E.gKh63**	Jackal	scox1-1 (KT697626)	snad1-1 (KT697629)	This study
**E.gKh75**	Dog	scox1-2 (KT033487)	snad1-1 (KT033488)	This study
E.gKh77	Dog	scox1-1 (KX186686)	snad1-1 KX186689)	This study
E.gKh86	Wolf	scox1-1 (KT697627)	snad1-1 (KT697630)	This study
E.gKh87	Dog	scox1-1 (KT697628)	snad1-1 (KT697631)	This study
E.gKh90	Jackal	scox1-1 (KX186687)	snad1-1 KX186690)	This study
E.gKh96	Dog	scox1-1 (KX186688)	snad1-1(KX186691)	This study
**E.mKh2**	Fox	scox1-3 (KT318127)	snad1-2(KT318129)	This study
**E.mKh4**	Jackal	scox1-3 (KT033486)	snad1-3 KT033489)	This study
E.mKh11	Fox	scox1-3 (KT318128)	snad1-2 (KT318130)	This study
E.mKh20	Jackal	scox1-3 (KX186692)	snad1-2(KX186699)	This study
E.mKh22	Jackal	scox1-3 (KX186693)	snad1-2 KX186700)	This study
E.mKh30	Jackal	scox1-3 (KX186694)	snad1-2 KX186701)	This study
E.mKh41	Jackal	scox1-3 (KX186695)	snad1-2 KX186702)	This study
E.mKh47	Jackal	scox1-3 (KX186696)	snad1-2(KX186703)	This study
E.mKh55	Jackal	scox1-3 (KX186697)	snad1-2(KX186704)	This study
E.mKh81	Jackal	scox1-3 (KX186698)	snad1-2(KX186705)	This study
*Echinococcus* genotypes/species				
G1^a^	Sheep	na	AJ237632	[12, 13]
G1^b^	Sheep	AF297617	AF297617	[69]
G2	Sheep	M84662	AJ237633	[12, 13]
G3^a^	Buffalo	M84663	AJ237634	[12, 13]
G3^b^	Sheep	DQ856466	DQ856469	[70]
G4	Horse	M84664	AJ237635	[12, 13]
G5	Cattle	M84665	AJ237636	[12, 13]
G6^a^	Camel	M84666	AJ237637	[12, 13]
G6^b^	Camel	NC-011121	NC-011121	[67]
G7^a^	Pig	M84667	AJ237638	[12, 13]
G7^b^	Goat	DQ856468	DQ856471	[70]
G8	Moose	AB235848	AB235848	[67]
G10	Reindeer	AF525457	AF525297	[71]
*E. felidis*	Lion	EF558356	EF558357	[66]
*E. multilocularis*^*a*^	Human	M84668	AJ237639	[12, 13]
*E. multilocularis*^*b*^	Rodent	M84669	AJ237640	[12, 13]
*E. shiquiqus*	Pika	AB208064	AB208064	[67]
*E. vogeli*	Rodent	M84670	AJ237641	[12, 13]
*E. oligarthrus*	Rodent	M84671	AJ237642	[12, 13]
Outgroup				
*T. saginata*	Cattle	na	AJ239106	[72, 73]

^a, b^Related to Fig. 7

*Note*: Representative haplotypes used in the phylogenetic analysis are indicated in bold

*Abbreviation*: na, not available

### Phylogenetic analysis

**Fig. 7:**
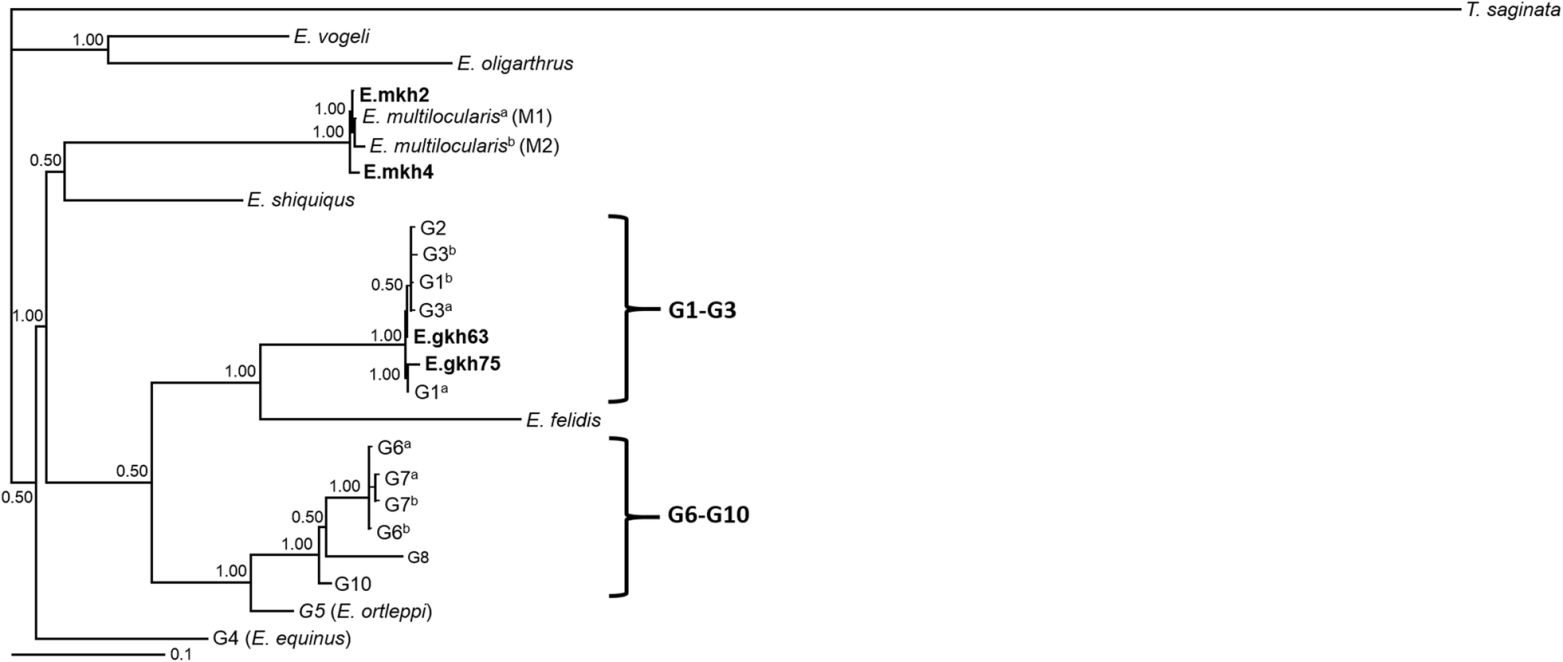
Phylogenetic tree of *Echinococcus granulosus* and *Echinococcus multilocularis* isolates from North-Khorasan Province, northeastern Iran (indicated in bold) and reference sequences for *E. granulosus* (*sensu lato*) and other species of *Echinococcus* chosen from previous studies. The relationships were estimated based on phylogenetic analysis of concatenated *cox*1+*nad*1 sequence data (600 nucleotides in total) using Bayesian inference; the sources and accession numbers of the sequences are listed in Table 3. The scale-bar indicates the number of substitutions per site. Nodal support is given as a posterior probability (pp) value. *Taenia saginata* was used as the outgroup

A rooted phylogenetic tree using *T. saginata* as the outgroup was constructed based on concatenated data of 600 nucleotides (nt) including *cox*1 (282 nt) and *nad*1 (318 nt) using Bayesian inference. Overall, two haplotypes of *E. granulosus* (*s.s*.), referred to as E.gKh63 and E.gKh75, and two haplotypes of *E. multilocularis*, referred to as E.mKh2 and E.mKh4, were observed in the present study. Representative haplotypes of *E. granulosus* clustered with a strong support (pp = 1.00) with G1–G3 genotypes, and representative haplotypes of *E. multilocularis* clustered with a strong support (pp = 1.00) with the reference sequences of *E. multilocularis* (Fig. 7).

## Discussion

Echinococcosis is one of the main neglected zoonotic diseases [50]. *Echinococcus granulosus* (*s.l.*) is known to exist in all continents, while *E. multilocularis* is displaying endemic districts in central Europe, northern and central Eurasia and particular parts in North America [2]. With regard to the Middle East, Iran is known as one of the endemic countries for both *E. granulosus* and *E. multilocularis* [34]. *Echinococcus granulosus* is highly prevalent throughout the country [34], and *E. multilocularis* is endemic in part of the territory in the north of the country, geographically located near Armenia, Azerbaijan, Turkey and Turkmenistan where *E. multilocularis* is endemic [35].

In Iran, in contrast to the large amount of epidemiology data available for *E. granulosus* [3], only a few studies have been performed on *E. multilocularis*. *Echinococcus multilocularis* was reported for the first time in 1971 in three of 30 red foxes (10%) in the Moghan Plain of north-western Iran [31]. Later, in 1992, a study in the Ardabil Province in northwestern Iran has shown that 22.9% of the red foxes and 16% of the jackals studied were infected with *E. multilocularis* [32]. The results of both studies were based on morphological identification of adult worms. In a subsequent study in 2009 on canine echinococcosis in Moghan Plain, using copro-PCR and CA-ELISA, *E. multilocularis* was not reported [51]. In a recent study, *E. multilocularis* was reported in carnivores in Chenaran City, Razavi Khorasan Province in the north-east of the country for the first time, using multiplex PCR targeting *nad*1 on DNA extracted from the stool samples of the hosts [33]. The present study was undertaken due to a lack of knowledge on the distribution of echinococcosis in definitive hosts in North-Khorasan Province, which is located on the expanded distributional range of *E. multilocularis* from the northwest to the northeast of the country. To our knowledge, this is the first study on *E. multilocularis* in Iran utilizing both morphological characteristics of rostellar hooks and strobila of adult worms as well as molecular analysis of two genomic regions, *cox*1 and *nad*1.

Overall, morphological descriptions of adult *Echinococcus* species and molecular identification of both mitochondrial genes were in agreement. For species identification based on light microscopy, the dimensions and shape of the rostellar large and small hooks were useful. With regard to the strobila, the position of the genital pores of proglottids and the shape of the uterus in the gravid segments were important distinguishing factors between the two *Echinococcus* species. However, in immature worms, there was an overlap in the size of strobila and proglottids of *E. granulosus* and *E. multilocularis*, which needs consideration during morphological analysis of adults of these two species to prevent misidentification.

Overall, 17 animals out of 106 examined (16%) had echinococcosis. The overall rates of infection with *E. multilocularis* and *E. granulosus* were 9.4% and 6.6%, respectively. In jackals, both *E. multilocularis* (13.1%) and *E. granulosus* (3.3%) were found. Infection of this canid with these species of *Echinococcus* has been reported on multiple occasions and across a wide geographical range [52]. In Iran, infections in jackals with *E. multilocularis* has been reported from Ardabil Province in the northwest [32] and Razavi Khorasan Province in the northeast of Iran [33]. In Hungary, the jackal was recently reported as a new host record for *E. multilocularis* [53]. The jackal is under significant and fast geographical expansion [52] and can migrate long distances through ecological corridors [53]. Since this species is an important definitive host for echinococcosis, it can serve as a potential source of infection to humans and domestic animals in endemic areas.

The other canids studied here were infected with either *E. multilocularis* or *E. granulosus.* In foxes, the overall infection rate of *E. multilocularis* was 8.7%, and in dogs, the overall infection rate with *E. granulosus* was 21%. Among the three wolves available for examination, one harboured *E. granulosus*. Among different canids examined in the Ardabil Province, northwest Iran [32], foxes were found infected with *E. multilocularis* while dogs and wolves harboured *E. granulosus*; these observations are in agreement with our findings. In Kazakhstan, which has one of the world’s largest wolf populations [54], 19.5% of evaluated wolves were observed to be infected with *E. granulosus*. Globally, *E. multilocularis* is widely prevalent among foxes [55–58] while *E. granulosus* appears much less abundant [59–62].

In dogs, although some studies reported the occurrence of *E. multilocularis*, the susceptibility of dogs to this species is estimated to be very low [3]. In the present study, the lack of *E. granulosus* in foxes and *E. multilocularis* in dogs and wolves may be due to the low sample sizes. Nevertheless, there seems to be ample evidence that foxes are more susceptible to *E. multilocularis* than *E. granulosus*, while for dogs and wolves it is *vice versa*.

In the present study, although all infected animals were males (17 out of 83) and none of the 23 female animals were infected, no statistical analysis was performed due to sex imbalance in the study sample. However, risk factors evaluation of echinococcosis in a highly endemic region of the Tibetan Plateau [63] indicated that male dogs were more likely to be infected than female dogs, based on univariate and multivariate logistic regression analysis (*P* < 0.01). This increased risk for male animals was attributed to the activity of maintaining territory and hunting, which is likely higher in male dogs compared with female dogs [63].

Molecular analyses of echinococcosis in Iran have been performed mostly on CE in humans and domestic animals [15, 19, 20, 22] and only to a limited extent on adult worms in the definitive hosts [24–27]. This difference mostly reflects difficulties related to field studies of definitive hosts, such as obtaining stray dogs and wild canids, contamination with viral infections (i.e. rabies) and high risk of hydatid cyst infection during examinations. Thus, the number of molecular studies on adult worms of *E. granulosus* in Iran is limited. To date, *E. granulosus* (*s.s.*) (G1–G3) and *E. intermedius* (G6) have been reported from Iranian canids. The first study in this respect appeared in 2012 in Lorestan Province, West of Iran, and involved genotyping 20 isolates of *E. granulosus* from dogs using sequencing of mitochondrial *cox*1 and *nad*1 genes. In that study, G1, G2 and G3 genotypes were reported [24]. In another survey in northwestern Iran, using the *cox*1 gene as a molecular marker, G1, G3 and G6 genotypes of *E. granulosus* were identified among 16 dogs [25]. Later, *E. granulosus* G1 and G3 genotypes were identified in dogs and G1 in jackals from the Caspian Sea, north of the country, using sequencing of the *cox*1 gene [26]. In the present study, which used both *cox*1 and *nad*1 genes, all seven *E. granulosus* isolates form canids of North Khorasan Province belonged to the G1 genotype. This is compatible with the results of all previous relevant studies in Iran and emphasizes G1 as the dominant genotype.

In the present study, the *cox*1 gene revealed more genetic diversity within the *E. granulosus* than within the *E. multilocularis* isolates. However, the *nad*1 gene showed a higher degree of sequence variation in *E. multilocularis* compared with *E. granulosus* isolates. Four representative haplotypes of this study along with reference genotypes/species of *Echinococcus* were included in the phylogenetic tree using Bayesian inference method. The phylogenetic tree indicated six different clusters for *Echinococcus* spp. As expected, G4 (*E. equinus*) and *E. shiquicus* were placed as two distinct clades. Two representative haplotypes of *E. multilocularis* isolates from our study (E.mkh2 and E.mkh4) and two geographical genotypes of *E. multilocularis* namely M1 (China, Alaska, North America) and M2 (European) formed a clade sister to *E. shiquicus* with maximum statistical support (pp = 1.00) [12, 13]. The distinct placement of *E. multilocularis* relative to *E. shiquicus* was poorly supported (pp = 0.50) but it is in concordance with some previous studies [19, 24, 64]. *Echinococcus vogeli* and *E. oligarthrus* formed a clade with high statistical support (pp = 1.00), which is in agreement with previous studies [7, 24, 64]. *Echinococcus felidis* was recovered as a sister taxon to G1–G3 genotypes in a distinct clade with maximum statistical support (pp = 1.00); this is in concordance with the studies of Lavikainen et al. [65], Hüttner et al. [66], Saarma et al. [7] and previous studies in Iran [15, 64]. Two *E. granulosus* haplotypes from the present study (E.gkh63 and E.gkh75) identified as the G1 genotype were grouped with reference G1 genotype within the cluster with G1–G3 genotypes with maximum statistical support (pp = 1.00). Our finding provides further evidence that G1–G3 genotypes are separate from other species or genotypes of *Echinococcus* and should be considered as *E. granulosus* (*s.s.*) [66, 67]. Furthermore, our data are in agreement with the statement that G2 is not a distinct genotype [8, 19, 68]. G6–G10 genotypes clustered separately from the G5 genotype within a clade with strong statistical support (pp = 1.00), which is in agreement with previous studies in Iran [15, 17, 26] and confirms reconstruction of the G5 genotype as *E. ortleppi* [11, 66, 67].

## Conclusions

In conclusion, our study confirmed that both *E. multilocularis* and *E. granulosus* are present in canines of the North-Khorasan Province, Iran. The distribution of *E. multilocularis* is wider than previously known. The jackal acts as a definitive host for both *E. multilocularis* and *E. granulosus*, but the infection rate with the former species is higher. This poses a potentially large risk of AE transmission to humans, especially in rural areas where jackals roam closer to human settlements than other wild canids. The increasing public health concern of this lethal zoonotic disease requires surveillance and early diagnosis of the infection in at-risk populations in the country. Future studies aiming to identify intermediate hosts for *E. multilocularis* in this endemic area of echinococcosis are needed. In addition, this area is suitable for further studies on the comparison of the biology of *E. granulosus* and *E. multilocularis*, with regard to infectivity across domestic and wild hosts.

## Data Availability

The data supporting the conclusions of this article are included within the article. The accession numbers of the sequences deposited in GenBank are provided in Table 3.

## Abbreviations

AE: alveolar echinococcosis
CE: cystic echinococcosis
*cox*1: cytochrome *c* oxidase subunit 1 gene
IST: intestinal scraping technique
*nad*1: NADH dehydrogenase subunit 1 gene
VDRC: Vector-Borne Diseases Research Center
